# Novel Approach for Isolation and Identification of Porcine Epidemic Diarrhea Virus (PEDV) Strain NJ Using Porcine Intestinal Epithelial Cells

**DOI:** 10.3390/v9010019

**Published:** 2017-01-21

**Authors:** Wen Shi, Shuo Jia, Haiyuan Zhao, Jiyuan Yin, Xiaona Wang, Meiling Yu, Sunting Ma, Yang Wu, Ying Chen, Wenlu Fan, Yigang Xu, Yijing Li

**Affiliations:** College of Veterinary Medicine, Northeast Agricultural University, Harbin 150030, China; wenshi_china@163.com (W.S.); jiashuo0508@163.com (S.J.); zhywxn1925@163.com (H.Z.); neauyjy@126.com (J.Y.); xiaonawang0319@163.com (X.W.); yu19890130@126.com (M.Y.); masunting@163.com (S.M.); wuyang_neau@126.com (Y.W.); chenying_neau@163.com (Y.C.); fanwenlu1230@163.com (W.F.)

**Keywords:** porcine epidemic diarrhea virus (PEDV) NJ strain, porcine intestinal epithelial cells, isolation and identification

## Abstract

Porcine epidemic diarrhea virus (PEDV), which is the causative agent of porcine epidemic diarrhea in China and other countries, is responsible for serious economic losses in the pork industry. Inactivated PEDV vaccine plays a key role in controlling the prevalence of PEDV. However, consistently low viral titers are obtained during the propagation of PEDV in vitro; this represents a challenge to molecular analyses of the virus and vaccine development. In this study, we successfully isolated a PEDV isolate (strain NJ) from clinical samples collected during a recent outbreak of diarrhea in piglets in China, using porcine intestinal epithelial cells (IEC). We found that the isolate was better adapted to growth in IECs than in Vero cells, and the titer of the IEC cultures was 10^4.5^ TCID_50_/0.1 mL at passage 45. Mutations in the S protein increased with the viral passage and the mutations tended towards attenuation. Viral challenge showed that the survival of IEC-adapted cultures was higher at the 45th passage than at the 5th passage. The use of IECs to isolate and propagate PEDV provides an effective approach for laboratory-based diagnosis of PEDV, as well as studies of the epidemiological characteristics and molecular biology of this virus.

## 1. Introduction

Porcine epidemic diarrhea (PED), which is caused by the porcine epidemic diarrhea virus (PEDV), is an acute and highly contagious enteric viral disease in nursing pigs. PED is characterized by vomiting and lethal watery diarrhea; and is a global problem, especially in many swine-producing countries [1,2,3,4,5,6,7]. PED was first reported in feeder pigs and fattening swine in the United Kingdom in 1971 [8]; since then, it has emerged in numerous European and Asian countries, resulting in tremendous economic losses to the pork industry worldwide. In 2013, the first PED outbreak was reported in the U.S.; subsequently, the outbreak spread rapidly across the country, and similar outbreaks were also reported in Canada and Mexico [4,5,6,7]. In China, PED outbreaks have occurred infrequently with only sporadic incidents [9,10]. However, in late 2010, a remarkable increase in PED outbreaks was reported in the pork-producing provinces [11,12]. In 2014, an outbreak of severe acute diarrhea, with high morbidity and mortality, occurred in sucking piglets in Nanjing, China. Herds vaccinated with the CV777-inactivated vaccine were also infected.

During this period, the effectiveness of the CV777-based vaccine was questioned as PED outbreaks also occurred in vaccinated herds [12]. PED has since become one of the most significant epidemics affecting pig farming in China [13]. PEDV is an enveloped, single-stranded, positive-sense RNA virus belonging to the genus *Alphacoronavirus*, family *Coronaviridae*, and order *Nidovirales* [14,15]. The size of its genome is approximately 28 kb, with 5′- and 3′- untranslated regions (UTRs) and seven open reading frames (ORFs) that encode four structural proteins, i.e., spike (S), envelope (E), membrane (M), and nucleocapsid (N), and three nonstructural proteins [10,16]. The S protein of PEDV is the major enveloped protein of the virion, associated with growth adaptation in vitro and attenuation in vivo [17]. In addition, the S glycoprotein is used to determine the genetic relatedness among PEDV isolates and for developing diagnostic assays and effective vaccines [18,19,20].

The ability to propagate the virus is critical for the diagnosis and molecular analysis of PEDV, particularly the development of inactivated or attenuated vaccine. However, propagation of PEDV in vitro is challenging. Even though PEDV may be isolated from clinical samples, it gradually loses its infectivity during further passages in cell culture [4]. Therefore, it is necessary to evaluate the disinfection efficiency in vitro viral isolates using a cell culture system that promotes growth of PEDV. Currently, several PEDV strains, such as CV777, KPEDV-9, and 83P-5, have been successfully propagated in Vero cells using media with added trypsin [21,22,23]. In recent years, new variants of PEDV have emerged that are difficult to isolate and propagate in Vero cells with trypsin. Researchers have attempted to use pig bladder and kidney cells to isolate PEDV, with the addition of trypsin to the medium; this is the first report of isolation of PED virus in porcine cell culture [24]. PEDV infects the epithelium of the small intestine, which is a protease-rich environment, and causes atrophy of the villi resulting in diarrhea and dehydration; this indicates that porcine intestinal epithelial cells are the target cells of this virus. In 2014, Wang et al. established a porcine intestinal epithelial cell line (ZYM-SIEC02) by introducing the human telomerase reverse transcriptase (hTERT) gene into small intestinal epithelial cells derived from a neonatal, unsuckled piglet [25]. Several studies have used this established porcine intestinal epithelial cell (IEC) line [26,27]; however, the characteristics of PEDV cultured in this cell line have not been reported.

The present study aimed to confirm and identify PEDV in samples collected from piglets with suggestive clinical signs, using the IEC line established by Wang et al. [25]. A PEDV isolate, named PEDV strain NJ, was successfully isolated. Our results show that the PEDV strain NJ is adapted to growth in IECs with media containing trypsin, suggesting a new approach for the propagation of PEDV. Furthermore, the phylogeny and mutations of the *S* gene during serial passages were analyzed to determine its genetic homology and molecular variability. A virulence experiment for IEC-adapted NJ also confirmed that the virus had a tendency towards attenuation at 45 passages.

## 2. Materials and Methods

All applicable international and national guidelines for the care and use of animals were followed. Approval (2016NEFU-315) was obtained from the Institutional Committee of Northeast Agricultural University for the animal experiments.

### 2.1. Cells and Clinical Samples

The swine intestinal epithelial cell (IEC) line established by Wang et al. was kindly provided by Prof. Yanming Zhang, College of Veterinary Medicine, Northwest A&F University, Yangling, Shaanxi, China. IEC and Vero cells (ATCC CCL-81) were cultured in Dulbecco’s modified Eagle’s medium (DMEM; Gibco, Grand Island, NY, USA), and supplemented with 10% fetal bovine serum (Gibco). The clinical samples (small intestine tissues) used in this study were collected from a pig farm in Nanjing, China, at which an outbreak of acute diarrhea among piglets had been reported. The virus isolated from samples was identified as PEDV by *M* gene-based reverse transcription PCR (RT-PCR). The small intestine tissue was homogenized with serum-free DMEM, and then centrifuged (Thermo Scientific Sorvall Legend Micro 17, Waltham, MA, USA) at 5000× *g* at 4 °C for 10 min. The supernatant was filtered using 0.22-μm pore-size cellulose acetate (Merck Millipore, Darmstad, Germany), and used for virus isolation.

### 2.2. RNA Extraction and RT-PCR Assay

Total RNA was extracted from the clinical samples and virus cultures using the TRIzol^®^ Plus RNA Purification Kit (Invitrogen Corp., Carlsbad, CA, USA) according to the manufacturer’s instructions. Complementary DNA (cDNA) was produced via reverse transcription using the Superscript Reverse Transcriptase Reagent Kit (Takara, Tokyo, Japan) according to the manufacturer’s instructions. The primer pairs of the partial *M* gene (316 bp) for identification of PEDV, 5′-TATGGCTTGCATCACTCTTA-3′ (forward) and 5′-TTGACTGAACGACCAACACG-3′ (reverse), were designed based on PEDV strain CV777, using the cDNA as a template. The PCR reaction system in a total volume of 50 μL was as follows: 5 μL of 10× buffer, 3 μL of cDNA, 1 μL of LA Taq polymerase (TaKaRa, Tokyo, Japan), 2 μL of forward primer (10 μM), 2 μL of reverse primer (10 μM), 4 μL of dNTPs mix-ture (2.5 μM), and sterile water added up to 50 μL. The cycling parameters for PCR included 95 °C for 5 min, followed by 30 cycles at 94 °C for 30 s, 55.5 °C for 30 s followed by 72 °C for 30 s, and a final extension at 72 °C for 10 min. The PCR purified products were cloned into pMD-19T vector and sequenced by Comate Bioscience Company Limited (Jilin, China).

### 2.3. Virus Isolation

In this study, Vero cells and IECs were used to propagate PEDV. For the propagation of PEDV using Vero cells, the confluent cell monolayer was washed once with sterile phosphate-buffered saline (PBS; pH 7.2), and incubated with 1 mL of inoculum for 1 h in a T25 flask supplemented with 21 μg/mL of trypsin (Gibco) at 37 °C under 5% CO_2_. Then, the inoculum was removed and the cells were washed twice with PBS, and 4 mL of maintenance medium (DMEM, Gibco) without fetal bovine serum supplemented with 5 μg/mL trypsin was added to the flask. The propagation of PEDV using IECs was performed according to the method described above, but with the use of 10 μg/mL of trypsin during adsorption. In parallel, cells mock-inoculated with DMEM were used as control. The PEDV infected cells and viral control cells were cultured at 37 °C under 5% CO_2_. The cytopathic effect (CPE) was monitored daily, and cells were harvested until the CPE exceeded 80%. After one freeze-thaw cycle, the supernatants were collected, packed separately, and stored at −80 °C until required. Virus titer was measured in 96-well plates by 10-fold serial dilution of samples at five-passage intervals. The 50% tissue culture infective dose (TCID_50_) was expressed as the reciprocal of the highest dilution showing CPE by the Reed–Muench method.

### 2.4. Electron Microscopy Assay

The supernatants of PEDV-infected IEC cultures were centrifuged at 3000× *g* for 45 min, followed by ultracentrifugation through a 25% sucrose cushion at 30,000× *g* for 2 h at 4 °C. Virus particles were resuspended in 100 μL of DMEM and observed by transmission electron microscopy (H-7650, Hitachi, Tokyo, Japan). For imaging of virions in infected IECs, PEDV-infected cells were fixed using 2.5% glutaraldehyde at 4 °C for 8 h, washed twice with PBS, and post-fixed with 2% osmium tetroxide at room temperature (20 °C–25 °C) for 50 min. After three washes with PBS, cells were dehydrated through a graded ethanol propylene oxide series and embedded. Then, ultra-thin sections were prepared and imaged via transmission electron microscopy.

### 2.5. Immunofluorescence Assay

After inoculation for 24 h, mock-infected IECs and IECs infected with PEDV, at multiplicity of infection (MOI) of 0.1 were fixed with 4% paraformaldehyde at room temperature (20 °C–25 °C) for 15 min, permeabilized with 0.2% Triton X-100 in PBS at room temperature (RT) for 10 min, and blocked with 0.3% bovine serum albumin in PBS at 37 °C for 30 min. Next, mouse anti-PEDV S protein monoclonal antibody (developed in our laboratory) and fluorescein isothiocyanate (FITC)-conjugated goat anti-mouse immunoglobulin G (IgG) (ZSGB-BIO, Beijing, China) were incubated as first and second antibodies, respectively, followed by counterstaining with 4,6-diamidino-2-phenylindole (DAPI, Beyotime, Shanghai, China). The coverslips were mounted on microscope glass slides in mounting buffer and cell staining was examined using a fluorescence microscope (Leica, Wetzlar, Germany).

### 2.6. Sequence Alignment and Phylogenetic Analysis of the S Gene

To monitor the amino acid variation of the S protein during the serial passaging, the parental NJ strain and the strain at different passages, i.e., NJ (15th), NJ (30th) and NJ (45th), were evaluated by RT-PCR. The *S* gene was amplified in four fragments using KOD-Plus-Neo (Toyobo, Osaka, Japan). The primers used were previously described by Zhao et al. [28]. The four fragments were amplified under same program of 2 min at 94 °C, 30 cycles of 10 s at 98 °C, 30 s at 52 °C and 1.5 min at 68 °C, and a final extension at 68 °C for 7 min. The purified PCR products were cloned into pMD-19T vector and sequenced by Comate Bioscience Company Limited (Jilin, China). The sequence analysis was performed using MegAlign in DNAStar Lasergene V 7.10 (DNAstar, Madison, WI, USA). To determine the relationships among the *S* gene of the representative PEDV isolates, phylogenetic analysis of the parent NJ strain was performed by the neighbor-joining method using molecular evolutionary genetics analysis (MEGA) software (version 4.0). Bootstrap values were estimated for 1000 replicates. The *S* gene sequences of PEDV strain NJ and the sequences of 33 known PEDV strains (listed in Table 1) retrieved from GenBank were subjected to comparative analysis.

### 2.7. Virulence Experiment for IEC-Adapted NJ

The in vivo swine studies were performed in a biosafety level-2 (BSL-2) laboratory. For the identification of attenuation, the pathogenicity of the lower and higher generations of NJ should be compared. PEDV NJ cultures propagated in IECs at passages 5 and 45 were used in this study. The PEDV-negative piglets as confirmed by RT-PCR method were neonatal landraces obtained from a pig farm without a PED outbreak or vaccination with PEDV vaccine. We randomly selected 20 healthy piglets as experimental animals. The piglets were divided into three groups. The two infected groups (eight pigs for each group) received an oral dose of 10^4.5^ median tissue culture infective dose (TCID_50_)/mL of IEC-adapted NJ (5 mL) at passages 5 and 45, and the control group (*n* = 4) was orally administered virus-free cell culture media. The clinical signs and survival percentage of the piglets were monitored daily over a 10-day observation period, and stool samples were collected daily. The small intestine tissue samples were collected and stored at −80 °C until required. RT-PCR and immunofluorescence assays were performed to detect PEDV in the stool samples. Necropsy was performed when the challenged piglets died post inoculation. The piglets were handled and maintained under strict ethical conditions according to international recommendations for animal welfare.

## 3. Results

### 3.1. Virus Isolation

**Figure 1:**
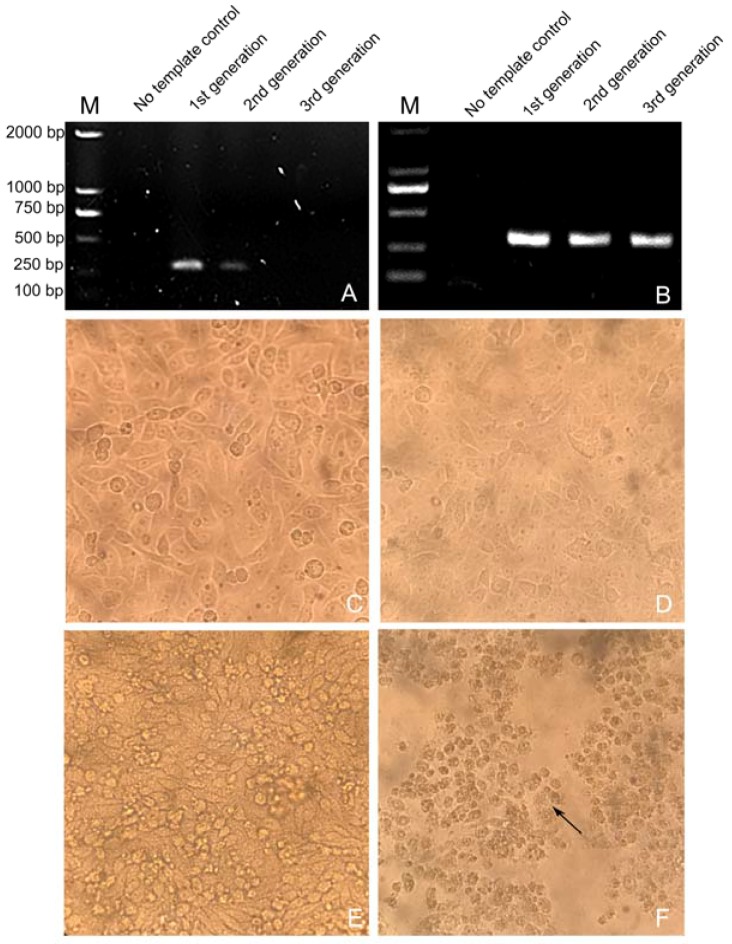
Isolation and identification of PEDV strain NJ in Vero cells and intestinal epithelial cell (IEC) cultures. (**A**) Identification of PEDV cultured in Vero cells at serial passages, by reverse transcription PCR (RT-PCR); (**B**) Identification of PEDV cultured in IEC at serial passages, by RT-PCR; (**C**) Control (uninfected) Vero cells; (**D**) PEDV-infected Vero cells; (**E**) Control (uninfected) IECs; (**F**) PEDV-infected IECs.

PEDV was isolated from PEDV-positive samples collected from pig farms in China, using Vero cells and IECs, in which a severe outbreak of acute diarrhea had been reported in sucking piglets. The genomic RNA of the serially propagated virus was extracted and identified by RT-PCR. Vero cell cultures were negative for the *M* gene after two passages (Figure 1A), and no CPEs were observed in Vero cells during serial passaging (Figure 1C,D). IEC-adapted PEDV was successfully propagated (Figure 1B), and visible CPEs were observed at each passage; compared with uninfected IECs, the PEDV-infected IECs were characterized by cell fusion, syncytial and vacuole formation in the initial stage, then shrinkage, detachment, and amotic at 72 h post-inoculation (Figure 1E,F). The virus cultured in IECs and designated NJ was biologically cloned by three rounds of plaque purification in IECs prior to further virus characterization. These results demonstrate that the adaption of the PEDV strain NJ to growth in IECs was better than that in Vero cells, indicating that IECs are suitable for the isolation of PEDV from clinical samples.

### 3.2. Determination of Viral Titer

**Figure 2:**
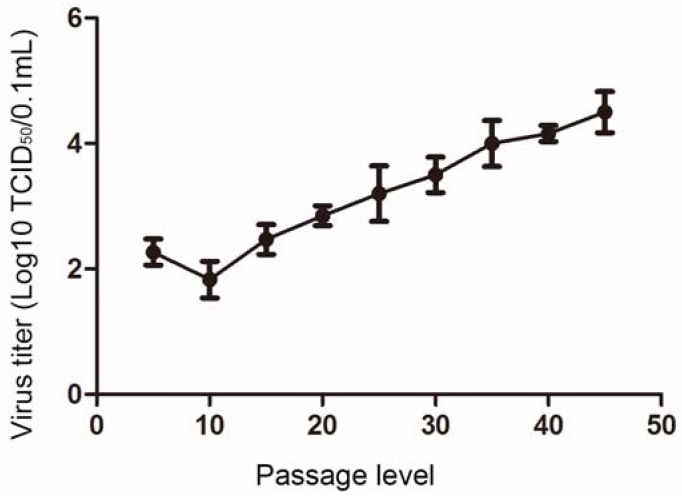
Viral titers of PEDV strain NJ propagated in IECs post-serial passages. All the results of a representative experiment performed with triplicate samples are shown.

The viral titer of the PEDV strain NJ propagated in IECs was determined at 5-passage intervals. The viral titer of the IEC-adapted PEDV strain NJ reached 10^4.5^ TCID_50_/0.1 mL at passage 45 (Figure 2), suggesting that the use of an IEC culture is a promising approach for propagating PEDV.

### 3.3. Electron Microscopy

**Figure 3:**
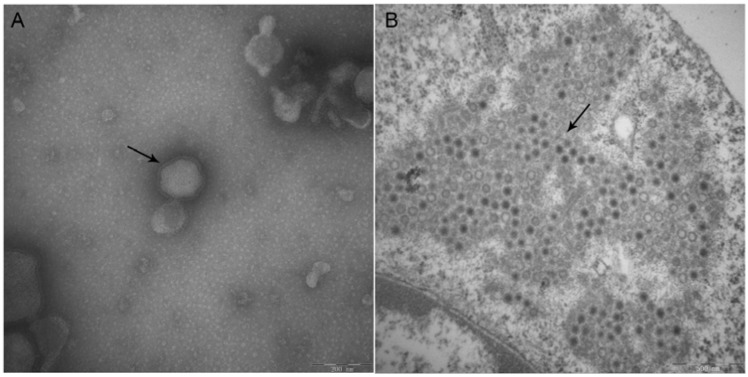
Images of PEDV strain NJ particles and PEDV strain NJ-infected IEC produced by electron microscopy. (**A**) Virions in culture media of IECs infected with PEDV strain NJ, as shown by the arrow; Bar = 200 nm. Magnification, ×100,000; (**B**) Thin section of IECs infected with PEDV strain NJ 24 h post-infection; many of the virus particles possessed a dense core and gathered in the cytoplasm as shown by the arrow; Bar = 500 nm. Magnification, ×50,000.

As shown in Figure 3A, the virion was circular in shape and 80–120 nm in diameter, with surface projections characteristic of coronaviruses. Thin sections of the PEDV strain NJ-infected IECs showed some of the virus particles appeared, many of the virus particles possessed a dense core, and masses of virus particles gathered in the cytoplasm at 24 h post-infection (Figure 3B).

### 3.4. Immunofluorescence

**Figure 4:**
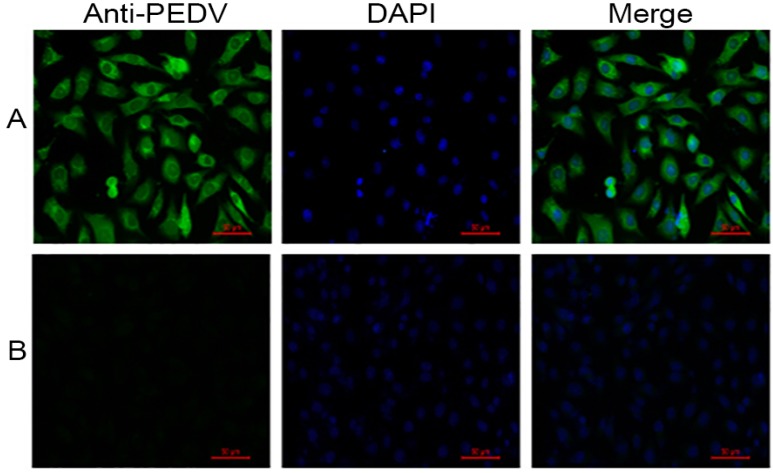
Detection of PEDV strain NJ in IECs by immunofluorescence assay at 24 h post-infection; mouse anti-PEDV S protein monoclonal antibody and fluorescein isothiocyanate (FITC)-conjugated goat anti-mouse immunoglobulin G (IgG) were respectively used as primary and secondary antibodies, followed by counterstaining with 4,6-diamidino-2-phenylindole (DAPI). (**A**) PEDV strain NJ-infected IECs; (**B**) Non-infected IECs.

Infection of IECs with the PEDV strain NJ was confirmed by immunofluorescence assay (Figure 4). PEDV strain NJ and cell nuclei were detected using mouse anti-PEDV S protein monoclonal antibody and 4,6-diamidino-2-phenylindole (DAPI), respectively. Specific green signals were observed in the PEDV strain NJ-infected IECs, but not in the mock-infected IECs. However, because the immunofluorescence assay was performed after inoculation for 24 h, the CPE was hard to observe during this period.

### 3.5. Phylogenetic Analysis of the S Gene

**Figure 5:**
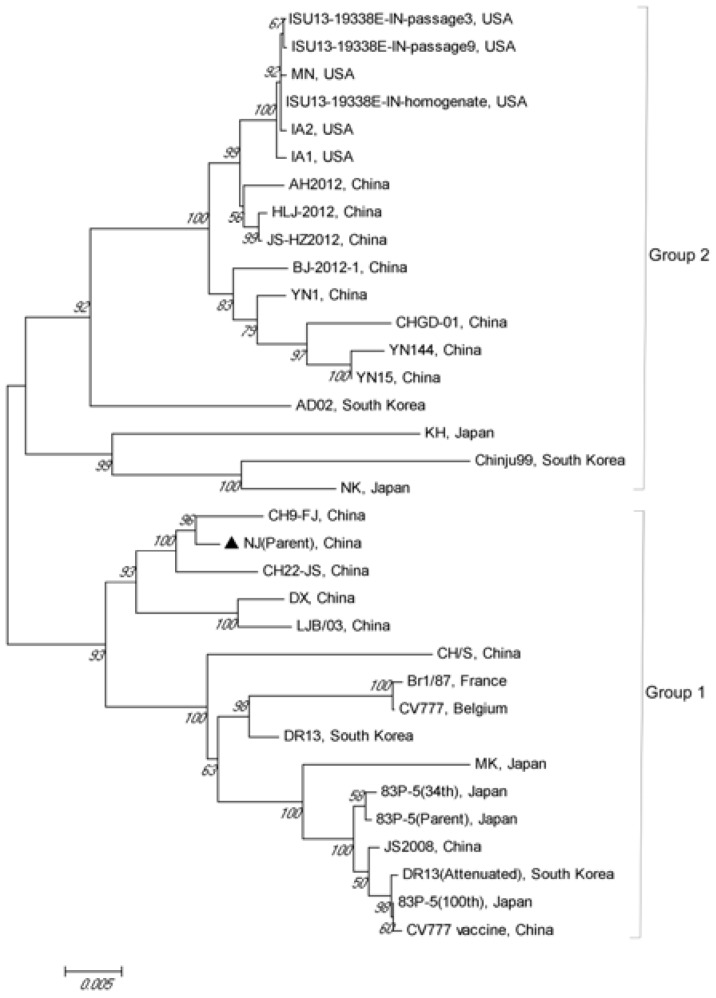
Phylogenetic analysis of PEDV strain NJ based on the *S* gene.

The *S* gene of PEDV strain NJ was amplified by RT-PCR. Phylogenetic analysis based on the *S* gene was performed between parental PEDV strain NJ and other PEDV strains listed in Table 1. Phylogenetic analyses of *S* gene sequences revealed that all PEDV strains in this study could be separated into two groups: the NJ strain belonged to Group 1, which also contained classical PEDV CV777 and some strains isolated from China, Japan, and South Korea. As shown in Figure 5, the *S* gene of the parent NJ strain exhibited high sequence similarity with the epidemic strains CH9-FJ, CH22-JS, and DX isolated from southern China in recent years. These results suggest that the Chinese southern epidemic PEDV strains were likely derived from the same source. In addition, we did not find any insertions and deletions (INDEL) in the *S* gene compared with the Chinese PEDV *S* gene recombinant variants like IA1, IA2, and MN identified in U.S.

### 3.6. Amino Acid Variability of the S Protein of IEC-Adapted NJ after Serial Passaging

To evaluate the amino acid variability of the IEC-adapted PEDV strain NJ during serial passagings, the sequences of the *S* gene of the parent NJ and those of the virus at the 15th, 30th and 45th passage were amplified by RT-PCR. These amino acid sequences were then compared with the corresponding S protein sequences of the classical PEDV CV777 and its vaccine strain. The positions of amino acid changes were 3, 15, 70, 114, 282, 324, 378, 438, 973, 1023 and 1167 respectively (Table 2). In total, four mutations occurred at passage 15, three and four more mutations occurred at passages 30 and 45. The eight (8/11) mutations were the same as the ones occurring in the transition from classical PEDV CV777 to the CV777 vaccine strain during serial passaging, which suggested that the NJ might exhibit a tendency towards attenuation.

### 3.7. Pathogenicity Analysis of PEDV Strain NJ

**Figure 6:**
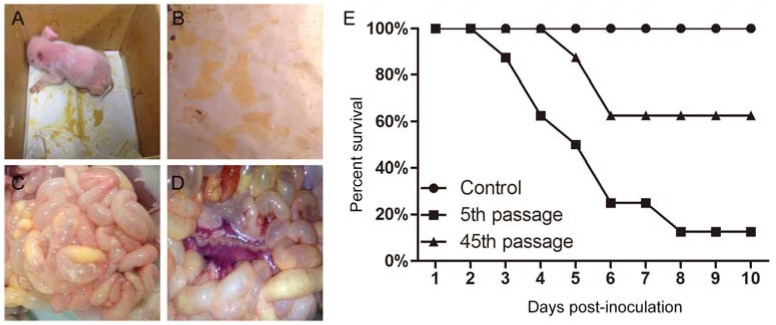
The clinical signs and necropsies of results of pigs infected with IEC-adapted NJ at 45th passage and the survival percentage of piglets after challenge. (**A**) Piglet with diarrhea and significantly dispirited status; (**B**) Watery diarrhea; (**C**) The intestinal tracts of infected piglets were thin and transparent; (**D**) Mesenteric congestion; (**E**) Survival percentage of piglets after challenge.

To compare the pathogenicity of the lower-generation NJ with that of the higher-generation NJ, 5 mL of the IEC-adapted NJ culture at the 5th and 45th passage (10^4.5^ TCID_50_/mL) and volume-matched virus-free cell culture medium were administrated orally to the piglets. The clinical signs and survival percentage of the piglets was monitored daily over a 10-day observation period. All piglets infected with 5th passage cultures and three (3/8) piglets infected with 45th passage cultures showed diarrheic feces, significantly emaciated body condition, and experienced severe watery diarrhea with vomiting. Abundant yellow, watery, and foul-smelling stools were also observed around the perianal region of the piglets infected with 45th passage virus (Figure 6A,B). The remaining five (5/8) piglets infected with 45th passage showed signs of mild diarrhea after inoculation that seemed to be transient. However, in the control group, the piglets were healthy without watery diarrhea (data not shown). As shown in Figure 6E, one piglet death occurred within 3 days and in total seven piglets died after the 10-day challenge in the group that received the 5th passage. However, only three piglets died in the group that received the 45th passage; although the remaining piglets had the significant clinical signs of PED, they survived the 10-day challenge. The small intestines of one of the dead piglets infected with 45th passage IEC-adapted NJ developed severe clinical symptoms, the same as those in the piglets infected with the 5th passage virus. The small intestines were typically distended with accumulation of yellow fluid and mesenteric congestion, and the small intestinal wall was thin and transparent (Figure 6C,D). The diarrheal feces and intestinal tissues collected from these piglets were analyzed by RT-PCR targeting the *M* gene, and the PCR products were found to be consistent with the expected result. Moreover, immunofluorescence analysis of the small intestine tissue revealed that the viral antigen was predominantly present in the small intestines (data not shown).

## 4. Discussion

The spread of PED to the U.S. and Canada has established that this viral infection represents a global epidemic [29] that has resulted in enormous economic losses to pig production. PEDV has caused similar economic losses to the pork industry in China, where frequent outbreaks have been recently reported [30]. Following the development of a CV777-based vaccine, based on the PEDV strain, and its wide application in the pig industry in China, only a limited number of incidents occurred before 2010; however, PED outbreaks have subsequently increased in frequency, particularly in pig-farming provinces. Notable, even pigs vaccinated with the CV777-based vaccine were found to be infected [31], indicating the need for the development of effective PEDV-based vaccines for the control of PED outbreaks.

To date, the propagation of PEDV remains challenging. Although Vero cells are commonly used to propagate PEDV, viral infectivity exhibits a gradual decline during serial passage in these cells. Many PEDV strains isolated from clinical samples were difficult to culture in Vero cells in recent years [4,32,33]. The adaptability of PEDV isolate infected with cell lines was the first step to successful isolation in vitro. Thus, it is urgent to develop new methods or cell lines to improve the tropism of PEDV cultured in cells. In this study, we successfully isolated and propagated the epidemic PEDV strain using porcine intestinal epithelial cells in vitro, and demonstrated that these cells are more suitable than Vero cells for the isolation of PEDV. This is the first report on the characteristics of PEDV cultured in this cell line which was established by Wang et al. [25].

The porcine intestinal epithelial cell is recognized as the target cell of PEDV [27]. Porcine aminopeptidase N (pAPN), a functional receptor of PEDV that is highly expressed in the small intestinal mucosa, plays a critical role in PEDV infection [31]. In addition, endogenous protease in the small intestine of porcine can cleave the S protein of PEDV in vivo and facilitate entry of PEDV virions into intestinal epithelial cells, resulting in massive propagation [34]. PEDV strain NJ isolated from clinical samples was as difficult to culture in Vero cells as other isolated prevalent PEDV strains; this is mainly attributed to mutations that arise in genes encoding spike proteins during serial passage. Moreover, the low levels of pAPN in Vero cells are thought to limit the attachment and entry of variant viruses. Before this study, we compared the isolation rate of five different positive PEDV strains isolated from clinical samples cultured in IECs and Vero cells. We found all the CPEs in Vero cells were invisible in three generations, and four PEDV isolates could not be detected by RT-PCR after two generations. In contrast, porcine IECs are more suitable than Vero cells for propagating PEDV isolated from clinical samples in vitro. The CPEs in IECs could be observed at the first or the third passage, and at each passage, cultures could be detected. Due to the characteristics of coronavirus, the degradation of the virus impacts the efficiency of the infection. The S glycoprotein forms peplomers on the virion envelope and contains receptor-binding regions and four major antigenic sites [35,36]; therefore, to minimize damage to the surface projections of the virus, the infected IECs were freeze-thawed only once before inoculation. The PEDV strain NJ was detected in cell culture during serial passages, by RT-PCR as well as immunofluorescence assay, indicating that this strain adapted to infection of IECs. Owing to the sensitivity of IECs, the trypsin concentration used in IEC culture was lower than that used in Vero cells during PEDV absorption. The viral titer of the IEC-adapted PEDV strain NJ propagated in IECs increased gradually and reached 10^4.5^ TCID_50_/0.1 mL at 45 passages.

The S protein of PEDV is known to play pivotal roles in viral entry and in inducing the neutralizing antibodies in natural hosts, which makes it a primary target for development of effective vaccines against PEDV [23,37,38,39]. In this study, the *S* gene of the PEDV strain NJ was amplified by RT-PCR, and its genetic diversity and phylogenetic relationships were analyzed. *S* gene-based phylogenetic analysis showed that the PEDV strain NJ is closely related to the CH9-FJ, CH22-JS, and DX strains isolated from southern China in recent years. In addition, we did not find any insertions or deletions (INDEL) in the *S* gene compared to the Chinese PEDV *S* gene recombinant variants like IA1, IA2, and MN identified in the U.S.

The differences in virulence and genome sequences between the parental strain and derived attenuated strains have been extensively studied [40,41]. To investigate the genetic variability of the S protein of the PEDV strain NJ, the S gene was amplified during various serial passages and analyzed. Compared with the parent NJ strain, four mutations in the S protein occurred after the 15th passage, and then another seven mutations occurred after the 30th passage. In total, 11 amino acids changed during 45 passages. The eight (8/11) mutations were the same as those occurring in the transition from the classical PEDV CV777 strain to CV777 vaccine strain during serial passaging, which suggests that the high-passage IEC-adapted NJ might show attenuation. However, the molecular basis of viral adaptation to IECs remains a subject for future investigation.

Furthermore, to identify attenuation, the pathogenicity was compared for the lower- and higher-generation NJ. The piglets were inoculated with 5 mL of the IEC-adapted PEDV strain NJ (10^4.5^ TCID_50_/mL) at the 5th and 45th passages. We found that all of the piglets infected with 5th passage IEC-adapted cultures showed severe watery diarrhea with vomiting, and seven (7/8) piglets died by day 10 post inoculation. However, only three (3/8) piglets infected with viral cultures at the 45th passage showed significant PED signs, such as diarrheic feces and emaciated body condition; the remaining five (5/8) piglets showed mild diarrhea after inoculation that seemed to be transient, and only three piglets died by day 10 post inoculation. The in vivo challenge experiment implied that IEC-adapted NJ at a high passage number had a tendency towards attenuation. The mutations in the S protein during serial passaging might play a major role in the attenuation of virulence, and might suggest potential genetic changes for a candidate attenuated vaccine. However, the genetic mutations in the whole genome of the NJ strain during passages needs to be investigated further.

In conclusion, we successfully isolated and identified a novel PEDV, strain NJ, from clinical samples using IECs. The adaption of the PEDV strain NJ to growth in IECs was better than that in Vero cells. To our knowledge, this is the first report to describe the isolation and characterization of the IEC-adapted PEDV strain NJ. Furthermore, the present work reveals a novel approach for the propagation of PEDV in vitro. These findings are expected to be of importance for the development and evaluation of the efficacy of vaccines against PED.

## Figures and Tables

**Table 1:** Reference strains of porcine epidemic diarrhea virus (PEDV) used in this study.

Strain	ID	Country	Strain	ID	Country
83P-5 (parent)	AB548618	Japan	HLJ-2012	JX512907	China
83P-5 (34th)	AB548619	Japan	IA1	KF468753	USA
83P-5 (100th)	AB548621	Japan	IA2	KF468754	USA
AD02	KC879281	South Korea	ISU13-19338E-IN-homogenate	KF650370	USA
AH2012	KC210145	China	ISU13-19338E-IN-passage3	KF650371	USA
BJ-2012-1	JX435299	China	ISU13-19338E-IN-passage9	KF650372	USA
Br1/87	Z25483	France	JS2008	KC109141	China
CH9-FJ	JQ979287	China	JS-HZ2012	KC210147	China
CH22-JS	JQ979290	China	KH	AB548622	Japan
CHGD-01	JN980698	China	LJB/03	DQ985739	China
Chinju99	AY167585	Korea	MK	AB548624	Japan
CH/S	JN547228	China	MN	KF468752	USA
CV777 vaccine	JN599150	China	NK	AB548623	Japan
CV777	AF353511	Belgium	YN1	KT021227	China
DR13	JQ023161	Korea	YN15	KT021228	China
DR13 (Attenuated)	JQ023162	Korea	YN144	KT021232	China
DX	JN104080	China			

**Table 2:** The amino acid variation of S proteins during the serial passaging compared with CV777 and its vaccine strain.

Strain	Amino Acid Position										
	3	15	70	114	282	324	378	438	973	1023	1167
NJ(Parent)	S	S	A	N	L	S	N	I	Y	K	A
NJ(15th)	S	S	D	N	L	S	N	I	H	N	D
NJ(30th)	A	S	D	S	L	S	K	I	H	N	D
NJ(45th)	A	L	D	S	W	R	K	L	H	N	D
CV777	S	P	A	N	L	S	N	I	Y	K	A
CV777 vaccine	P	L	D	S	W	F	K	V	H	N	D
